# Facilitated Effects of Closed-Loop Assessment and Training on Trans-Radial Prosthesis User Rehabilitation

**DOI:** 10.3390/s25175277

**Published:** 2025-08-25

**Authors:** Huimin Hu, Yi Luo, Ling Min, Lei Li, Xing Wang

**Affiliations:** 1 Department of Biomedical Engineering, Chongqing University, Chongqing 400044, China; huhuimin0912@163.com (H.H.); luoyii@stu.cqu.edu.cn (Y.L.); binghenmeng@gmail.com (L.M.); 2 Department of Biomedical Engineering, Faculty of Engineering, Hong Kong Polytechnic University, Hong Kong SAR, China; li-lei.li@connect.polyu.hk; 3 The Key Laboratory of Biorheological Science and Technology, Ministry of Education, Bioengineering College, Chongqing University, Chongqing 400044, China

**Keywords:** myoelectric prosthesis, closed-loop rehabilitation, virtual reality, multimodal assessment

## Abstract

(1) Background: Integrating assessment with training helps to enhance precision prosthetic rehabilitation of trans-radial amputees. This study aimed to validate a self-developed closed-loop rehabilitation platform combining accurate measurement in comprehensive assessment and immediate interaction in virtual reality (VR) training in refining patient-centered myoelectric prosthesis rehabilitation. (2) Methods: The platform consisted of two modules, a multimodal assessment module and an sEMG-driven VR game training module. The former included clinical scales (OPUS, DASH), task performance metrics (modified Box and Block Test), kinematics analysis (inertial sensors), and surface electromyography (sEMG) recording, verified on six trans-radial amputees and four healthy subjects. The latter aimed for muscle coordination training driven by four-channel sEMG, tested on three amputees. Post 1-week training, task performance and sEMG metrics (wrist flexion/extension activation) were re-evaluated. (3) Results: The sEMG in the residual limb of the amputees upgraded by 4.8%, either the subjects’ number of gold coins or game scores after 1-week training. Subjects uniformly agreed or strongly agreed with all the items on the user questionnaire. In reassessment after training, the average completion time (CT) of all three amputees in both tasks decreased. CTs of the A1 and A3 in the placing tasks were reduced by 49.52% and 50.61%, respectively, and the CTs for the submitting task were reduced by 19.67% and 55.44%, respectively. Average CT of all three amputees in the ADL task after training was 9.97 s, significantly lower than the pre-training time of 15.17 s. (4) Conclusions: The closed-loop platform promotes patients’ prosthesis motor-control tasks through accurate measurement and immediate interaction according to the sensorimotor recalibration principle, demonstrating a potential tool for precision rehabilitation.

## 1. Introduction

China has approximately 3 million amputees, with trans-radial amputations being the most common. Myoelectric prostheses play a vital role in restoring users’ independence; however, high abandonment rates remain due to unintuitive control, insufficient sensory feedback, and the lack of comprehensive functional assessment protocols [1,2,3]. Although myoelectric prosthetic technology has advanced significantly in recent years [4], their routine utility faces great challenges such as weak surface electromyography (sEMG) [5], updating training data in machine learning, limb-position effect [6], and dissatisfaction in train protocol. Hence, modern accurate assessment and immediate feedback of training is particularly critical for prosthetic rehabilitation, faced with new requirements such as dynamic assessment, increased training efficiency, online testing as new assessment standard resulted from rapid application of wearable sensors and information technology [7].

The increasingly popularity of kinematic metrics have exerted a huge influence on prostheses assessment. In addition to traditional subjective questionnaires such as the Orthotics and Prosthetics Users Survey (OPUS) [8] and the Disabilities of the Arm, Shoulder, and Hand Questionnaire (DASH) [9,10], kinematic tools enable process-based evaluation during tasks based on inertial measurement unit (IMU) sensors and motion capture cameras [11,12,13,14,15]. Hence, in accordance with the International Classification of Functioning, Disability and Health (ICF), assessment scales and questionnaires [16], such as AM-ULA, BBT, and the Quick DASH questionnaire [17] were utilized in a comprehensive manner. However, traditional assessment tools lack real-time trajectory recording in process, hindering precision assessment design. The richer kinematic data acquired from the wearable sensor laid the foundation for the possible process evaluation, which enables the development of intelligent assessment systems. The assessment of compensatory strategies during prosthetic tasks (e.g., trunk tilt in Box and Block Test, BBT) that leads to biased functional scoring [18,19] becomes possible.

Modern technology including wearable sensors and information processing has played a profound effect on prosthetic training. The advancement of prosthetic limb function prompts amputees to typically spend more time and effort in extensive training for better confidence in their use [20]. In traditional hospital-based rehabilitation, patients perform motor imagery exercises based solely on physicians’ instructions, without intuitive visualization of training outcomes or confirmation of movement execution. As a result, the effectiveness of the training cannot be assessed in real time. Virtual reality (VR) technology has emerged as a promising tool for prosthetic rehabilitation training, leveraging immersive environments and interactive design to enhance user engagement and motor relearning [21,22]. Early VR systems primarily focused on augmenting visual feedback to facilitate motor adaptation, exemplified by virtual versions of standardized assessments such as the BBT [23] and Southampton Hand Assessment Procedure (SHAP) [24]. However, these training systems often lacked a dynamic link between assessment and training, limiting their ability to dynamically adjust task difficulty based on real-time user performance. Recent advancements have integrated sensor technologies (e.g., IMUs) and motion capture devices (e.g., Kinect) enabling posture-aware VR training [25].

Despite recent innovations in both either assessment or training modules supported by wearable sensor and information technologies, there remains a significant gap in achieving precision rehabilitation for myoelectric prosthesis users, largely due to the patient-centered nature of myoelectric prosthetic care [26]. Precision rehabilitation, derived from precision medicine, emphasizes more accurate functional measurements, more frequent assessments, and the strategic use of technological advances such as electronic health records and wearable devices [27]. While traditional evaluations often rely on offline metrics (e.g., post-hoc classification accuracy of sEMG [28]), such approaches hinder real-time optimization of training protocols [29]. Comprehensive assessment and the motor recovery trajectory in training need to be integrated to develop patient-specific interventions [30]. Markus Nowak et al. proposed a simultaneous assessment and training protocol for myoelectric control that not only involved completing prosthetic tasks but also continuously monitored task progress, which enabled on-demand updating of prosthetic control programming in a machine-learning based device [31]. However, in contrast, non-machine learning prostheses—though more affordable and widely available—still lack integrated modules for assessment and training. In our previous work, Hu et al., we once built a multivariate assessment platform of a trans-radial prosthesis to provide a kind of multifaceted evaluation [32]. This study builds upon that platform by incorporating an interactive training module to form a closed-loop system. Is it possible that a self-developed closed-loop assessment–training–reassessment platform, integrating multifaceted kinematic and EMG-based interactive VR training on a set of self-developed platforms, facilitates motor-control training performance for non-machine learning prosthetic users? Does the unified assessment–training platform contribute to the precision rehabilitation of myoelectric prosthesis users as a feasible tool?

To verify this hypothesis, this study aims to develop a comprehensive and practical assessment–training–reassessment framework to improve the functional evaluation and training efficiency of trans-radial prosthesis users through a precision rehabilitation framework. The system integrates outcome-based metrics (e.g., task completion time (CT), game scores) and process-based indicators (e.g., kinematics, compensatory strategies), supported by a VR-based sEMG interactive training module. By conducting evaluations before and after the training phase and boosting motor learning through visual stimulation and sEMG control in VR games, the system augments a closed loop (assessment–training–reassessment) that enables continuous adjustment of muscular control and regulation of motor nerve centers.

## 2. Materials and Methods

The methodology of this study consists of three primary components:

- Initial assessment of prosthetic function;
- sEMG training of stump muscles;
- Reassessment of prosthetic function.

Additional components include the subjects, the experimental apparatus, and the experimental protocols.

### 2.1. Initial Assessment of Prostheses Function

The overall assessment framework comprises six components: sEMG assessment, clinical assessment, dexterity, coordination, motion smoothness, and self-assessment—covering the full range of ICF domains.

#### 2.1.1. Subjects

Six subjects with incomplete forearms (44.50 ± 12.75 years) and four healthy subjects (20.75 ± 0.83 years) were recruited for this study through the Rehabilitation Department of Southwest Hospital and the Disability Federation, as summarized in Table 1. One subject had a congenital incomplete forearm, whereas five had amputations due to trauma; among them, one underwent right-sided amputation and five had left-sided amputations with varying levels of limb loss. Only one amputee had utilized a prosthesis post-amputation. Subjects did not receive any additional treatments or rehabilitation during the study period. All had normal vision and reported no other medical conditions.

Prior to the commencement of the experiment, all amputee subjects were equipped with prosthetic sockets to enhance the stability and functionality of the prosthetic devices. The experimental protocol was approved by the Ethics Committee of the Three Gorges Hospital of Chongqing University (approval no. 2021-KY-24). All subjects provided written informed consent prior to participation in the study.

#### 2.1.2. Experimental Setup

**Figure 1:**
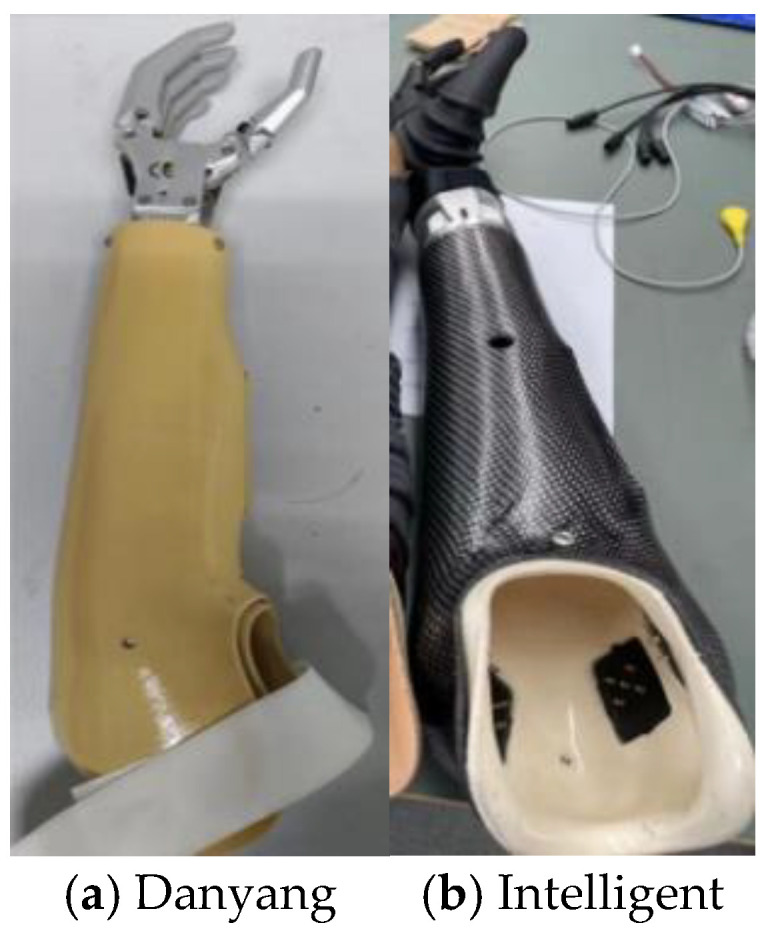
The trans-radial prosthesis used for the experiment.

The prostheses utilized in this experiment include a double-degree-of-freedom (DOF) trans-radial prosthesis (SJQ22, Danyang Prosthetic Factory, Danyang City, Jiangsu Province, China) and an intelligent prosthetic hand designed and developed by Shanghai Jiao Tong University. The former offers hand grip and wrist movement as its two degrees of freedom. In contrast, the latter possesses multiple degrees of freedom, including wrist flexion and extension, wrist internal and external rotation, and multiple hand degrees of freedom. Prosthetic adaptation was individualized based on residual limb length, muscle condition, and coordination capacity. The trans-radial prostheses used in the experiment are illustrated in Figure 1, and detailed specifications are provided in Table 2. Additionally, the experiment employed devices such as boxes, wooden blocks, water cups, and wooden pegs.

#### 2.1.3. Experimental Procedure

**Figure 2:**
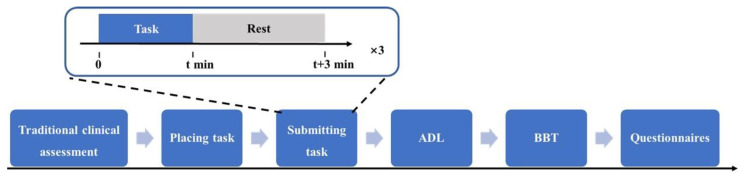
Paradigm of the initial prosthetic function assessment. The process began with a clinical assessment, followed by three repetitions of the placing and submitting task, then 10 ADL tasks, and finally five BBT trials assessing fine motor skills, followed by two questionnaires on experience with the prosthesis.

The experimental procedure shown in Figure 2 was expected to last approximately 1.5 h. To ensure effective operation, all participants underwent basic pre-assessment training that focused on simple control tasks such as hand opening and closing via residual forearm wrist flexion and extension movements enhanced by motor imagery, as well as muscle site identification through repeated practice.

The procedure formally commenced with a conventional clinical assessment to verify proper prosthesis fitting. Prosthesis size and weight were required to meet standard limits (≤1 cm length difference, ≤0.5 kg weight), and socket stability was evaluated by applying vertical traction, permitting no more than 2 cm displacement without causing skin discoloration.

This was followed by a dexterity assessment comprising three trials of the placing and submitting tasks to evaluate performance stability [33]. Prior to the experiment, two MPU6050 accelerometer and gyroscope modules (fire technology, 3-axis accelerometer, and 3-axis gyroscope) were affixed to the subject’s shoulder and elbow joints, with the sampling rate of 125 Hz, gyroscope measurement range of ±2000 deg/s, and the accelerometer measurement range of ±2 g. A digital low-pass filter with a cutoff frequency of 5 Hz was applied to the sensor data to reduce noise. The placing task evaluated the amputee’s ability to use the prosthetic hand to transfer objects between locations, while the submitting task assessed their ability to retrieve an item from the contralateral hand using the prosthesis. Subsequently, subjects executed ten distinct activities of daily living (ADL) tasks, dedicating one attempt to each. Based on prior pilot studies and the actual task performance of subjects, these ten ADL tasks were divided into simple tasks and complex task according to difficulty levels referring previous experiment [32]. Subsequently, five trials of the Box and Block Test (BBT) were conducted to evaluate fine motor skills. Finally, subjects completed two questionnaires designed to evaluate their subjective experience with prosthetic usage, encompassing comfort, satisfaction, and psychosocial factors. An association analysis will be performed upon study completion.

#### 2.1.4. Measurement Analysis

Objective results included task metrics and kinematic data, such as the CT for the placing task, the submitting task, and the ten ADL tasks, as well as the number of blocks moved in the BBT. Task CT was a key metric used to evaluate prosthetic control performance [33]. Movement smoothness was evaluated by calculating the rate of change, curvature, and ROM of the acceleration curves during the amputated subjects’ movement.

Subjective results were derived from both the task and prosthesis-related questionnaire and the NASA-TLX questionnaire [34]. The former (Q1–Q11) employed a 5-point Likert scale to assess subjects’ perceptions of task appropriateness, satisfaction, and comfort, as well as their evaluations of prosthetic weight, functionality, and comfort. The latter evaluated the actual impact of prosthetic design and functionality on users by measuring the psychological and physiological workloads during prosthetic use.

The Wilcoxon signed-rank test was conducted to examine differences, with the significance level set at *p* < 0.05. Descriptive statistics methods were applied to analyze the subjective results, aiming to better understand the sample characteristics, data distribution, and potential relationships among research variables.

### 2.2. sEMG Training of Stump Muscles

#### 2.2.1. Subjects

Three subjects with incomplete forearms were recruited for the sEMG training experiment, including A1 (congenital left incomplete forearm, 26 years old), A3 (traumatic left amputation, 39 years old), and A4 (traumatic left amputation, 61 years old), all of whom participated in the previous experiment. All subjects had no prior experience with the stump muscle sEMG training system, had normal vision, and did not have any other medical conditions. Details regarding ethical approval and informed consent for the experiments have been provided in the previous sections.

#### 2.2.2. Experimental Devices

During the experiments, an NI acquisition card (NI USB-6001, National Instruments, Austin, TX, USA), sEMG sensors (ZTEMG-1000, Qingdao Zhituo Intelligent Technology Co., Ltd., Qingdao, China), two PCs, and a multi-channel physiological signal acquisition and processing system for measuring the sEMG (RM6240, Chengdu Instrument Factory, Chengdu, China) were utilized. A PC1 (AMD Ryzen, 2 GHz, 8 GB, 14 inches), equipped with LabVIEW 2018 (National Instruments Corporation, USA), was responsible for real-time sEMG acquisition, signal visualization, and threshold setting for wrist flexion and extension, where the thresholds were determined by extracting the RMS values of the sEMG signals; PC2 (Intel^®^ Core i7 -7700HQ, 2.30 GHz, 16 GB, 15.6 inches) received commands and controlled the movement of the virtual game character.

#### 2.2.3. Experimental Procedure

**Figure 3:**
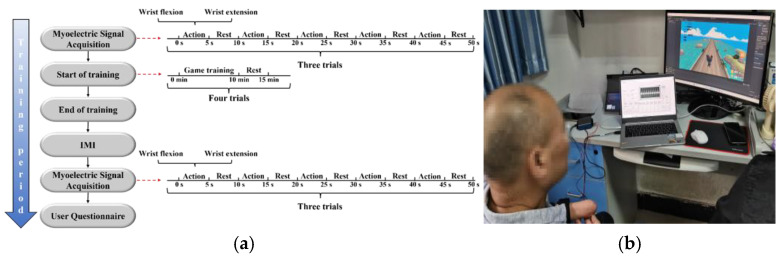
Paradigm and setup of sEMG-based training experiment: (**a**) Experimental paradigm for sEMG training. (**b**) Scene diagram of the training experiment.

The experimental procedure is shown in Figure 3 and was expected to take 1.5 h, which was like the study by Prahm’s team [35].

The experimental procedure followed these steps: (1) Disposable ECG electrodes (Hangzhou Xunda Radio Equipment Co., Ltd., Hangzhou, China) were used to collect sEMG from the subjects’ wrist flexion and extension movements before training. (2) After the original sEMG was collected, two EMG sensors were placed on the subject’s wrist flexor and extensor muscles (ensuring that the placement matched the original collection sites) and fixed with a bandage to ensure that the sensors were in close contact with the skin. (3) The RMS thresholds of the wrist flexion and wrist extension sEMG are adjusted, so that the sEMG signals from wrist flexion controlled the left-direction movement of the game character, and the sEMG of wrist extension controlled the right-direction movement of the game character. (4) Entering the learning phase of the game control, the subjects practiced reaching the “Left” and “Right” levels ten times each and then practiced controlling the sEMG to reach the “Right” level for ten additional times, which took about 10 min. (5) During the game training phase, the subjects controlled the sEMG to make the game object avoid obstacles and pick up gold coins, and the number of deaths were recorded. Each training trial lasted for 10 min, followed by a 5 min rest period. A total of 4 training trials were conducted per day, four times a week. The Intrinsic Motivation Inventory (IMI) was completed at the end of training. (6) At the end of 4th training in a week, sEMG during wrist flexion and wrist extension movements was recorded from the subjects, and a user questionnaire was filled out. (7) On the 2nd day after the whole training session, wrist flexion and extension sEMG were collected again to assess retention.

#### 2.2.4. Measurement Analysis

Objective measures include sEMG data, game scores, and the number of gold coins collected during gameplay. Subjective measures include ratings from the IMI scale and user questionnaires. sEMG data were processed using MATLAB R2022b (MathWorks, Inc., Natick, MA, USA) with a sampling rate of 1000 Hz and a 20–150 Hz Butterworth bandpass filter for removing noise and a 50 Hz notch filter to suppress power line interference. After preprocessing, signals were segmented into 5 s windows with 5 s steps for feature extraction. The mean RMS values of these segments were computed as key features. For game scores, gold coins, and subjective measures, statistical analysis was performed using Origin 2022 (Originlab, Inc., Northampton, MA, USA). The two questionnaires were the Intrinsic Motivation Scale [36] and the User Questionnaire. The former utilized a seven-point Likert scale and included five subscales including interest/fun, cognitive ability, tension/stress, cognitive choice, and value/utility, and it was completed at the end of each training session. The latter, completed after the final training, used a five-point Likert scale and encompassed the following questions: (1) Do you consider the system to be highly efficient? (2) Do you find the training with this system to be highly engaging? (3) Do you believe that training with this system is more effective? (4) Do you find the operation of the training system to be seamless? (5) Do you consider the difficulty level of the system to be appropriately challenging? (6) Are you interested in continuing training with this system?

### 2.3. Reassessment of Prosthetic Function

The reassessment procedure conducted after the training session, which was like the initial assessment but excluded traditional clinical evaluations, was designed to last approximately 1.5 h. Dexterity was first assessed, followed by a verification of movement stability through the execution of the placing and submitting tasks, each repeated three times. Subsequently, participants individually performed each of the ten activities of daily living (ADL) tasks. Finally, five Box and Block Test (BBT) trials were administered to evaluate fine motor performance.

## 3. Results

### 3.1. Assessment of Prosthetic Function

#### 3.1.1. Objective Indicators

- Traditional clinical assessment

The results of the traditional clinical assessment are shown in Table 3. All subjects’ trans-radial prostheses weighed over 0.5 kg, although the other results were good.

- Task Measurement Indicators

**Figure 4:**
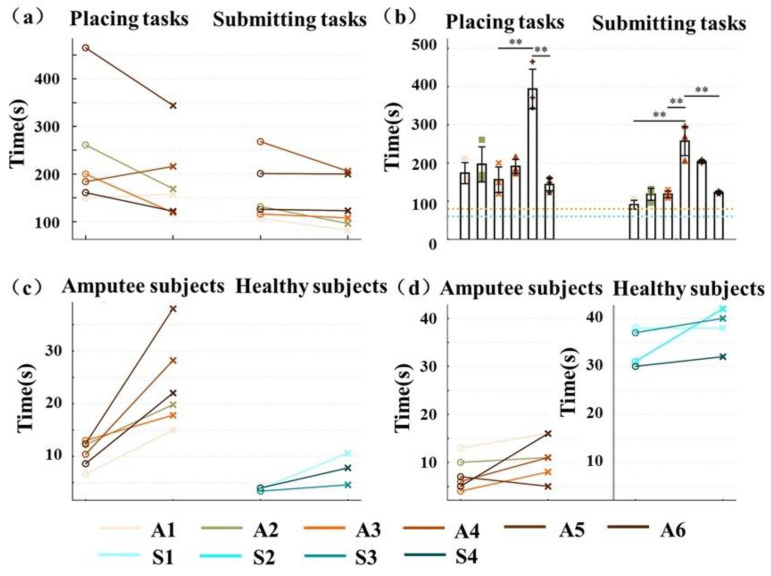
Results of the objective measures: (**a**) results of the placing and submitting task, with circles and forks representing the first and third trials, respectively; (**b**) bar charts of the placing and submitting tasks performed by six amputee participants, with the orange and blue dashed lines representing the standard time spent on the submitting task and the placing task, respectively; and (**c**) results of the ADL task, with circles and forks representing the average completion time (CT) for the simple task group and the complex task group, respectively; (**d**) results of the BBT task, with circles and forks representing the first and fifth trials, respectively. *Note:* ** indicates *p* < 0.01.

The results of the task measurement are shown in Figure. 4. In the placing and submitting tasks (Figure 4a,c), the third trial required significantly less time than the first trial. The amputation subjects’ task CT (Figure 4b) significantly exceeded the Chinese national yardstick [33]. The task CT increased with task difficulty (Figure 4c). In the BBT task (Figure 4d), subjects’ task CT also increased with the number of experimental trials. For moving 16 wooden blocks, amputees required approximately 1 min, whereas healthy subjects needed only about 30–40 s. In addition, there was a significant variation in the mean CT among amputee subjects across the five repeated experiments.

- Assessment of Motion Smoothness

**Figure 5:**
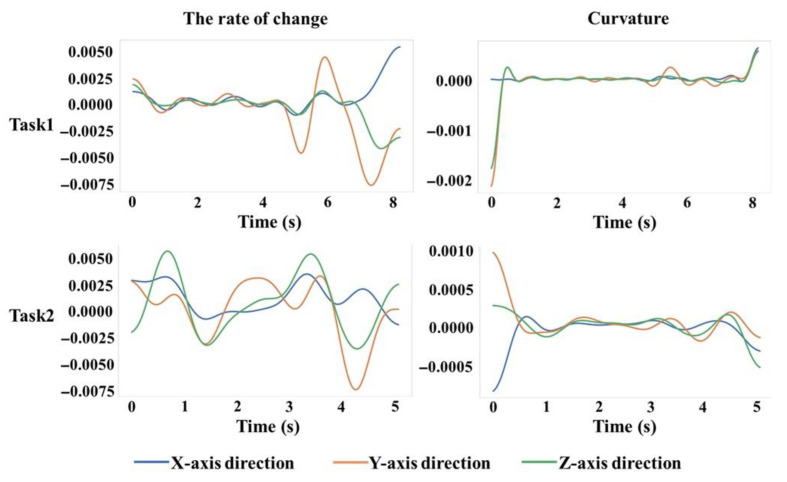
Plot of the rate of change and curvature of acceleration data during task A3.

As shown in Figure 5, sudden peaks in the rate of change of acceleration and curvature curves indicate that the subject exhibited significant movement at specific moments. To evaluate movement smoothness, comprehensive analyses were conducted across all participants. For illustrative purposes, the acceleration profile of Subject A3 was selected due to the relatively complete muscle data and participation in subsequent training sessions. During Task 1, the rate of change was initially stable but later exhibited significant fluctuations, indicative of reduced smoothness. Nonetheless, the curvature profile remained consistent, suggesting a generally smooth motion. The acceleration rate of change profile of Task 2 continued to fluctuate, demonstrating a lower degree of smoothness. In summary, Task 1 (complex task, pouring water) displayed greater movement smoothness compared to Task 2 (simple task, moving lighter objects).

**Figure 6:**
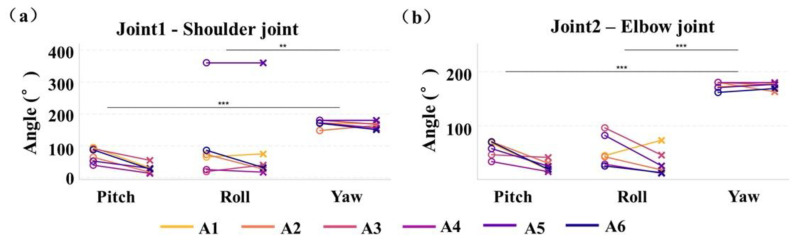
Range of motion during Task 1 and moving a light object: (**a**) range of motion of the shoulder joint of the amputee subject in both tasks; (**b**) range of motion of the elbow joint of the amputee subject in both tasks. Distinct colors represent individual subjects, with circles and forks denoting Tasks 1 and 2, respectively. *Note:* ** indicates *p* < 0.01, *** indicates *p* < 0.001.

The ROM results for the three directions/axes (x, y, and z)/(pitch, roll and yaw) during Task 1 and Task 2 are shown in Figure 6, which (a) illustrates the shoulder joint ROM and (b) presents elbow joint data. The data indicated that the shoulder joint ROM spanned from 0 to 200°, and the elbow joint ROM extended from 0 to 180°. Notably, the z-axis ROM for A5’s shoulder joint exhibited an outlier, ranging from 300 to 400°. In summary, the ROM was significantly greater in the y-axis than along the x and z axes.

#### 3.1.2. Subjective Indicators

**Figure 7:**
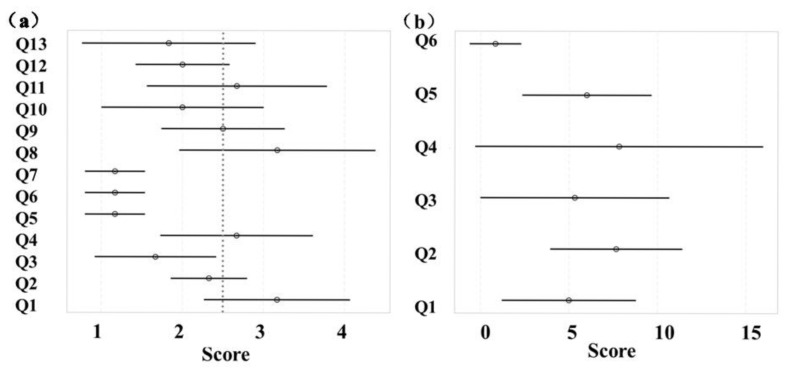
Summary of subjective scores: (**a**) subjective results for task and prosthetic satisfaction; (**b**) subjective results for overall task workload (NASA-TLX), which depicts median (circle) and maximum/minimum (line) values.

In Figure 7, the task setting and trans-radial prostheses function were rated below 2.5 (lower the score indicate higher satisfaction). In Figure 7b, the mean scores of the amputee subjects were below 10 on the NASA-TLX questionnaire, indicating lower mental, physical, and temporal demands, as well as reduced overall workload. However, scores related to prosthesis comfort (Q11) and weight (Q8) showed greater variability across subjects and between different tasks.

#### 3.1.3. Association Analysis

**Figure 8:**
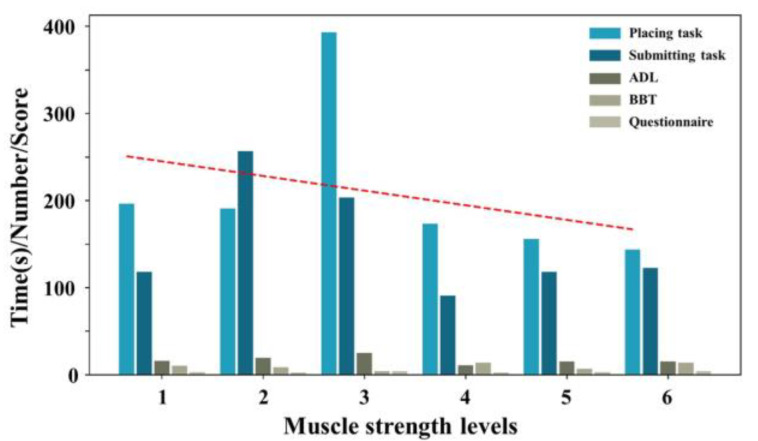
Bar chart of task performance metrics and muscle strength. We categorized the muscle strength of amputees from weak to strong as 1–6, representing A2, A4, A5, A1, A3, and A6, respectively. The red dotted line represents the fitted curve. The slope shows the change in myoelectric game (or score) induced by an increase in EMG signals along the x-axis.

Based on the outcomes presented in Figure 8, there is notable consistency among the assessment results, indicating that this method can yield outcomes analogous to those of muscle strength, thereby suggesting its reliability. The protracted CT for A5 and a significant rise in questionnaire scores may be attributable not only to diminished muscle strength but also potentially to advanced age and reduced comprehension abilities.

### 3.2. Stump Muscle sEMG Training

#### 3.2.1. Objective Indicators

- Mean value of RMS of sEMG signal

**Figure 9:**
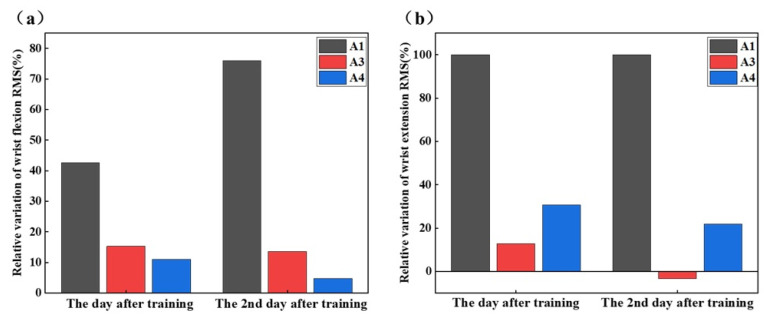
Overall changes in EMG RMS values during wrist movements before training, after training, and on the 2nd day post-training.(**a**) wrist flexion and (**b**) wrist extension.

According to Figure 9, the RMS mean values of sEMG in wrist flexors and wrist extensors increased by at least 4.8% on the day after training relative to the pre-training period. On the second day after training, the RMS averages of each subject exhibited varying trends. The RMS values of sEMG on the day after the training and on the second day after the training increased overall compared with pre-training.

- Game score and number of gold coins

**Figure 10:**
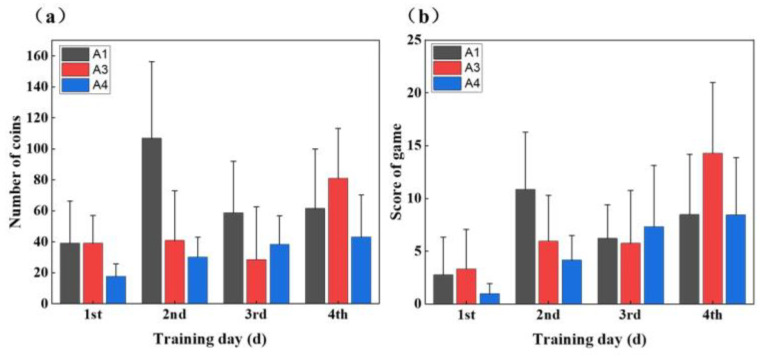
(**a**) Number of gold coins and (**b**) game score during training.

As shown in Figure 10, the subjects’ game performance (both the number of gold coins and game score) increased with increasing training days. Regarding these measures, the three subjects exhibited varying performances, with Subject A1 showing a significant increase on the second day, Subject A3 experiencing a decrease on the third day, and Subject A4 demonstrating a gradual upward trend.

#### 3.2.2. Subjective Indicators

- IMI Scale

**Figure 11:**
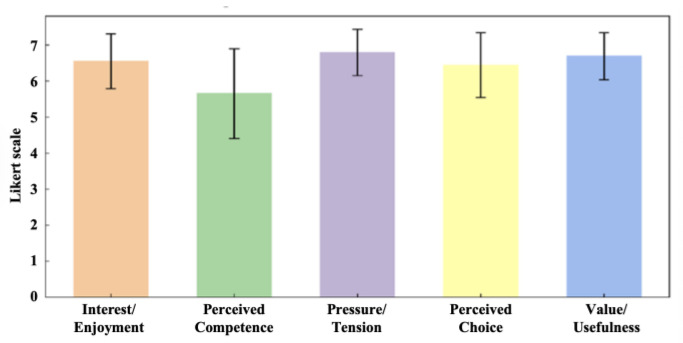
Results of the five subscales of the Intrinsic Motivation Scale. (Error bars indicate the standard deviation of the subscales.).

As depicted in Figure 11, the IMI scale results indicated that subjects exhibited a high level of interest in the game training and perceived it as their autonomous choice. Throughout the training, subjects reported feeling relaxed and acknowledged the system’s value and utility. However, the mean cognitive ability score was notably low (5.65 ± 1.25) when considering the subscale averages.

**Figure 12:**
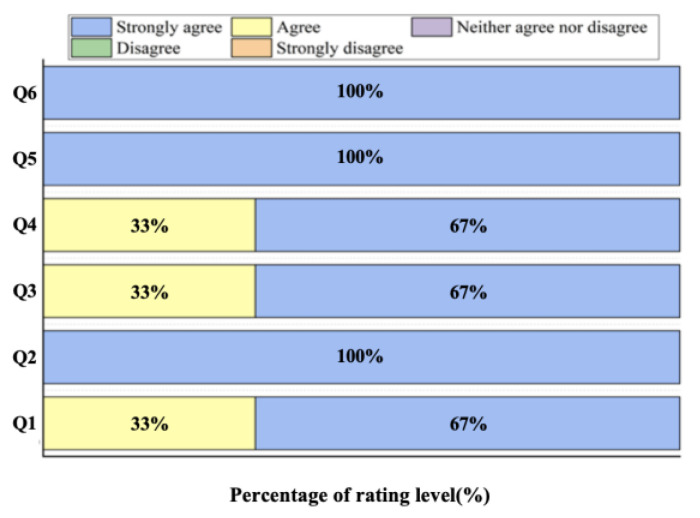
Results of the user questionnaire.

Based on the user questionnaire (refer to Figure 12), subjects consistently agreed or strongly agreed with all survey statements, indicating the system’s utility, engagement, operational fluency, and appropriate complexity. Additionally, they expressed a willingness to re-engage with the system for further training.

### 3.3. Reassessment of Prosthetic Function

#### 3.3.1. Placing Task and Submitting Task

**Figure 13:**
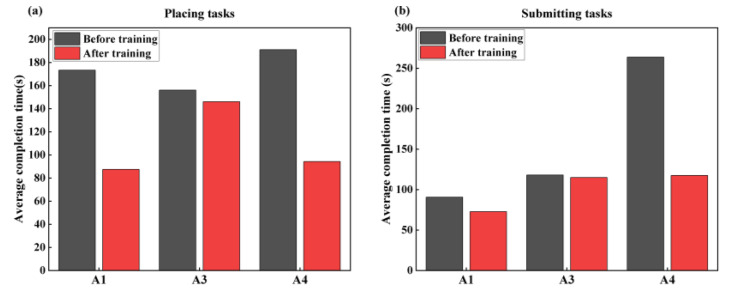
Comparison of completion time before and after training for different tasks. (**a**) Placing task and (**b**) Submission task.

The average CT for both the placing task and the submitting task (refer to Figure 13) decreased after training, although it remained higher than the Chinese national yardstick time. Notably, A1’s CT (72.83 s) in the submitting task met the national yardstick after training. Specifically, the post-training CTs of A1, A3, and A4 for the placing task were reduced by 49.52%, 6.41%, and 50.61%, respectively, and their CTs for the submitting task were reduced by 19.67%, 2.54%, and 55.44%, respectively.

#### 3.3.2. ADL and BBT

**Figure 14:**
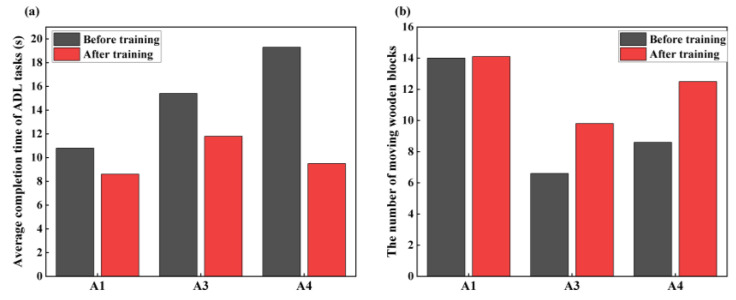
Results of pre- and post-training comparisons of ADL task and BBT task measures. (**a**) CT of ADL tasks. (**b**) The number of moving wooden blocks.

As shown in Figure 14a, the average CT of the ADL task after training was 9.97 s, significantly lower than the pre-training time of 15.17 s. Specifically, A3 and A4 had greater decreases in ADL CT (23.38% and 50.78%) than A1 (20.37%). As shown in Figure 14b, in the BBT task, the mean number of blocks moved by subjects in 60 s increased compared to the pre-training period. A1 demonstrated the smallest increase in the number of blocks moved (0.1 blocks), whereas A4 demonstrated the largest increase (3.9 blocks). Overall, A1’s performance was superior to that of the other subjects.

## 4. Discussion

### 4.1. Assessment of Prosthetic Function

The traditional clinical assessment results (Table 3) indicated that it was the length and the stability, rather than the weight, of the trans-radial prostheses that were suitable for the amputee subjects. The drop in CT along with the rising trials (see Figure 4) reflected that practice improved the operation speed in both wooden peg tasks. In addition, the shorter CT for the submitting task (Figure 4b) compared to that for the placing task is contrary to the Chinese national yardstick (GB/T 41841-2022 [33]). The cylindrical shape of the wooden pegs makes them more prone to sliding during the placing task than during the submitting task.

These differences may be attributed to the different amputation periods and sEMG levels of the amputee subjects. The shorter CT of A3 and A6 are due to either more balanced muscle strength, experience with prosthesis use, and a shorter duration since amputation. The longer CT of A4 and A5 (*p* < 0.05) indicated weaker movement coordination resulting from the imbalance of muscle strength of A4 and older age of A5. A similar trend was observed in the ADL and BBT tasks (Figure 4c,d) and the wooden peg tasks, for instance, with the consistently poorer performance of A4 and A5.

The relatively stable rate of motion change for A3 during the complex task (Figure 5) could be attributed to changes in task barriers or execution strategies. Complex tasks usually require more attention and motor control than simple tasks; therefore, A3 may have needed more adjustments to maintain motor smoothness during execution. The greater ROM observed in the complex task (Figure 6) could be attributed to the central nervous system’s regulation of prosthetic movement and its control over the joint mobility to accommodate the demands of the complex task. A5’s large ROM in the joint1 z-direction in the complex task may be due to individual physiological differences and challenges in prosthetic adaptation. NASA-TLX questionnaire results suggest that prosthetic design and materials require further improvement.

This comprehensive design of six components distinguishes our study from previous work [8,17,37,38], since previous studies typically included fewer dimensions. The multifaceted measurement laid the foundation for precision rehabilitation [27]. Notably, a preliminary analysis of compensatory behavior was performed, indicating that movements of the shoulder and elbow joints may partially reflect the extent of participants’ compensatory strategies.

In the initial assessment phase, subjects displayed variability in task performance, influenced by factors such as prosthesis type, muscle condition, and individual background. These inter-individual differences—often overlooked in standard clinical assessments—underscore the importance of personalized evaluation strategies. Our observations also support previous findings that highlight the role of compensatory strategies in functional performance scoring [16,17], further demonstrating the value of sensor-based analysis.

### 4.2. Stump Muscle sEMG Signal Training

The increase in RMS of both wrist flexors and wrist extensors (Figure 9) after training was probably due to the enhanced muscle adaptation. However, the differences observed on the day after training, during which sEMG signal continued to increase in A1, whereas they decreased in A3 and A4, may be related to physiological conditioning during fatigue and recovery. The overall trend showed an increase in RMS values after training and on the second day after training, verifying the training’s impact on sEMG signals. The lowest RMS values in A1 may have originated from the congenital limb hypoplasia, resulting in more severe muscle atrophy [39]. The decrease in RMS values for A3 on the second day after training may have stemmed from the incomplete recovery of muscle or fatigue accumulation.

A1 showed a significant increase in both game score and number of gold coins on the second day (Figure 10), probably due to his acquisition of basic skills and strategies during the first day. In contrast, A3’s number of gold coins and score in the game declined on the third day, likely attributable to the poor stump muscle condition caused by fatigue or insufficient rest, which affected the stability of the sEMG and the accuracy of the action recognition. A4’s performance was consistently stable and increased daily, which might be attributed to a more effective training method or strategy that enhanced his in-game proficiency.

Subjects concurred that the system’s training elicited positive physiological responses, aligning with Lohse’s findings [40]. Cognitive ability scored the lowest out of the five subscales (Figure 11), which could be attributed to the game’s high level of difficulty as well as the subjects’ lack of confidence in their cognitive abilities. To ameliorate this issue, incorporating hints and clear instructions into the game could facilitate comprehension and problem-solving for subjects. In addition, in-game music and immediate feedback can enhance training motivation [41]. In contrast, virtual gold rewards increase player engagement, resulting in higher scores on other subscales.

The results of the user questionnaire (Figure 12) reveal that the system achieved good results. After training with using the system, the muscle control of amputees improved significantly. The increased number of training trials gradually encouraged subjects’ game performance. In addition, the IMI scale and the user questionnaire confirmed the subjects’ positive recognition of the system and their willingness to continue participating.

In summary, the VR-based sEMG training system for amputees demonstrated significant effectiveness in improving muscle control and offered a viable and practical approach to rehabilitation. Compared with previous systems, it provided a more immersive and motivating user experience, effectively addressing the monotony commonly associated with traditional rehabilitation. The strong alignment between user-reported satisfaction (IMI and questionnaire results) and objective functional improvements further supports the feasibility and user-centered value of such VR-based myoelectric training approaches.

### 4.3. Reassessment of Prosthetic Function

A notable reduction in task CT and an increase BBT scores confirmed the effectiveness of our training system. These improvements, especially among subjects with lower baseline capabilities, suggest that the VR + sEMG system facilitates both neuromuscular adaptation and the transfer of skills to real-world prosthetic tasks. While some subjects’ progress was limited by prosthesis design or fatigue, the overall trend supports the value of adaptive training informed by continuous assessment.

The average CT reduction of 37% for the placing task among three subjects (Figure 13a) verified that the stump muscles’ sEMG training led to notable improvements in prosthetic control and task performance efficiency. However, the post-training CT remained above the Chinese national yardstick (GB/T 41841-2022), implying that longer training durations may be required to achieve further performance gains. Moreover, the elevated CT of A1 and A4 may stem from the Danyang prosthesis with fewer DOFs and simpler control. In contrast, the limited improvement observed in A3 may be due to the use of a multi-DOF intelligent prosthesis with greater functional complexity and more movement categories. Therefore, a multi-degree-of-freedom prosthesis may require multi-degree-of-freedom sEMG training and training time.

The average 35.36% reduction in CT (Figure 13b) for the three subjects was due to the training. A1 and A4 showed more substantial reductions in CT than other subjects in the submitting task. A1, likely due to his younger age and superior comprehension before training, adapted and learned more quickly after training, enabling the submitting task CT to meet the Chinese national yardstick.

Contrary to the flexibility assessment, in the ADL task (Figure 14a), the decrease in task CT for A3 and A4 exceeded that of A1. This may be explained by the fact that A1 had already performed well before training and had limited promotion space. Conversely, A3 and A4, who exhibited average performance pre-training, demonstrated significant enhancements post-training. Additionally, the decrease in task CT for A4 was more significant than that for A3, a difference potentially linked to prosthetic factors.

In terms of the number of blocks moved, A1 exhibited a smaller increase than A3 and A4 (Figure 14b), likely due to similar factors. The comparable improvements observed in A3 and A4 may be explained by their similar cognitive capacities, learning rates, and strategy adoption during the training process. In conclusion, stump muscle sEMG training with the VR system significantly upgraded the subjects’ prosthetic control and enhanced performance on various tasks, showing the positive effects of training on muscle control and neural adaptation.

To sum up, this study developed a closed-loop “assessment–training–reassessment” paradigm to systematically evaluate and enhance the functional use of trans-radial prostheses. Compared to previous systems [23,24,25,26,27,28], our framework seamlessly integrates training and assessment within a unified platform, which assists clinicians in better designing and adjusting training protocols, thereby laying a solid foundation for future implementation of personalized and real-time training adaptations. This proposed design effectively addresses key limitations in conventional prosthesis rehabilitation, including the lack of closed-loop learning, insufficient kinematic data, and the disconnection between training and assessment. So, this is the necessary significance of unified assessment and training, due to the patient-centered nature of prosthetic rehabilitation [26].

Despite these promising preliminary results, several limitations need future exploration. First, the training duration was relatively short—only one week—which may not adequately capture the long-term rehabilitation effects. Future work should extend the training period to assess the persistence of functional gains over time. Second, the current protocol was not fully personalized; future research may explore using initial assessments to guide individualized training programs, with subsequent reassessments to evaluate their effectiveness. Additionally, to enhance the adaptability of the system, future research should consider integrating more physiological signals—such as electroencephalography (EEG)—to support dynamic, real-time modulation based on users’ cognitive and attentional states.

## 5. Conclusions

This study significantly facilitated prosthetic control tasks in amputees through a closed-loop assessment–training–reassessment design. The closed-loop design platform proposed in this study is more rational and effective than a traditional discrete prosthesis assessment and training system. The unified assessment–training platform will contribute to the precision rehabilitation of myoelectric prosthesis as a feasible tool. The results of this study offer a valuable reference for improving prosthetic effectiveness in amputees and hold positive implications for future rehabilitation research and clinical practice.

## Figures and Tables

**Table 1:** Basic characteristics of amputee subjects.

Subject	Age	Gender	Amputation Status	Causes of Amputation	Prosthetic Type
A1	26	female	left arm	inborn	Danyang
A2	57	male	right arm	contraindication 27 years ago	Intelligent
A3	39	female	left arm	contraindication 32 years ago	Intelligent
A4	61	male	left arm	contraindication 43 years ago	Danyang
A5	51	male	left arm	contraindication 26 years ago	Intelligent
A6	33	male	left arm	contraindication 13 years ago	Intelligent

**Table 2:** Trans-radial prosthetic information.

Prosthesis Type	Hand Side	Degrees of Freedom	Control Method	Power Supply
Danyang	Left	2 (Hand open/close, wrist flexion/extension)	Dual-channel sequential control	8V lithium battery
Intelligent	Left/right	Multiple (multi-DOF hand, wrist flexion/extension, rotation)	16-channel pattern recognition control	8V lithium battery

**Table 3:** Traditional clinical assessment results.

Subject	Prosthetic Limb Size (Within 1 cm Shorter Than the Healthy Subject)	Prosthetic Limb Weight (≤0.5 kg)	Stability of the Prosthesis
A1	√	×	√
A2	√	×	√
A3	√	×	√
A4	√	×	√
A5	√	×	√
A6	√	×	√

## Data Availability

The data presented in this study are available on request from the corresponding author.
